# Editorial: Modern approaches to hemophilia management in children and adolescents

**DOI:** 10.3389/fped.2023.1273639

**Published:** 2023-09-06

**Authors:** Ali Amid

**Affiliations:** BC Children’s Hospital, Division of Pediatric Hematology/Oncology, Department of Pediatrics, University of British Columbia, Vancouver, BC, Canada

**Keywords:** hemophilia, thrombosis, children, bleeding, surgery

**Editorial on the Research Topic** Modern approaches to hemophilia management in children and adolescents

Hemophilia A and B are the most common severe bleeding disorders which are due to partial or total deficiency of coagulation factor VIII (FVIII) or factor IX (FIX), respectively. Depending on the severity of coagulation factor deficiency, the affected individuals may experience bleeding only with severe trauma or during major surgery (mild hemophilia) to spontaneous hemarthrosis, hematomas, and intracranial hemorrhages in the absence of any identifiable trauma (severe hemophilia) (1).

Until recently, coagulation factor replacement therapy via plasma-derived factor concentrates or recombinant factor products has been the mainstay of hemophilia treatment, provided either prophylactically or on-demand to treat bleeding episodes. Despite the widespread availability of safe and effective replacement therapy, many individuals with hemophilia continue to experience significant burden from their treatment, breakthrough bleeding, and debilitating arthropathy. In addition, the relatively high rate of inhibitor development to factor products can cause major morbidity and mortality, leading to a considerable burden on patients, families, and healthcare systems (2).

Over the past two decades, however, there has been remarkable progress in the management of hemophilia. Cloning of the genes encoding factor VIII and factor IX, together with advances in bioengineering have led to novel therapies that offer easier modes of administration, extended half-lives, and improved patient outcomes and quality of life. In addition, gene therapy is under investigation as the next frontier in hemophilia treatment, especially for hemophilia B (3, 4).

While these advances are associated with improvement of patients' outcomes, they also result in many new observations and questions that were not previously encountered. Furthermore, access to these treatments remains limited to the minority of people with hemophilia worldwide.

In this Research Topic, Urasiński et al. review the Polish national experience of switching prophylaxis factor replacement therapy from standard-dose prophylaxis with a plasma-derived FVIII to recombinant FVIII in previously-treated patients (PTP), as the result of the national tender. Authors aimed to address two important questions: safety of switching from a plasma-derived product to a recombinant product, and the effectiveness of PK-tailored dosing vs. standard-dose approach. While the national tender mandated the switching of the products, children and adolescents had the choice of standard-dose or PK-tailored approach. Following the switch, no patient developed high-titer or persistent inhibitor (two patients had low-titer but transient inhibitors). Curiously, patients who chose standard-dose prophylaxis had a lower bleeding rate prior to product switch as compared to those who elected to change the dosing regimen to a PK-tailored approach. This discrepancy likely indicates the sense of satisfaction in those who did not experience frequent bleeding with their historic standard-dose approach. However, after the switch, both groups achieved comparable bleed control, with more improvement in bleeding rate observed in those who selected the PK-tailored approach. As a drawback the consumption of rFVIII was higher in patients on PK-tailored prophylaxis. The study by Urasiński et al. is valuable as it offers its observations in a real-world data and in a unified national approach. While it is not reported in this study, it would be valuable to understand patient-reported outcomes (i.e., quality of life and patient satisfaction) using either standard-dose vs. PK-based approach.

In the second article, Belletrutti et al. review the literature and offer their approach for the management of surgeries in children and adolescents with hemophilia A who are undergoing surgeries while they are on emicizumab, a novel non-factor product. Emicizumab has dramatically reduced frequency of bleeding in patients with and without inhibitors. However, in children with Hemophilia A who require surgical or other invasive procedures, additional treatment with factor replacement or other hemostatic agents may still be needed to prevent intraoperative or postoperative bleeding. These scenarios can be quite challenging for clinicians, as excessive treatment can be associated with cost, and possibly, complications, while under-treatment are associated with undesired bleeding. Currently, there are no standard guidelines for preparing children with Hemophilia A on emicizumab for surgical procedures. In this review, Belletrutti et al. highlight the dilemma to offer factor replacement therapy prior to minor surgeries in children and adolescents with hemophilia A, as emicizumab may provide enough FVIII equivalent levels to allow for adequate hemostasis following low-risk procedures. Furthermore, they highlight the dilemma that may arise from surgeries in those who started emicizumab as their initial prophylactic treatment and prior to receiving any factor replacement therapy.

In the next review article in this special topic, El Maamari et al., offer an evidence-based approach for management of thrombosis in children with hemophilia, especially with the availability of more effective treatments (e.g., extended half life factor products or novel rebalancing agents). Indeed, as these options offer significant improvement of hemostasis, they carry an inherent increased risk of thrombosis. Both thromboembolic events and thrombotic microangiopathy have been observed as complications. These events can pose significant challenges as anticoagulation is a particularly risky endeavor in children with hemophilia. El Maamari et al., use clinical vignettes to review scenarios that clinicians may encounter and offer their approach. This review highlights the importance of an individualized approach and careful review of risk of bleeding vs. risk of thrombosis. Clearly, more high-quality data are needed to offer a robust approach while preventative measures remain paramount to reduce the risk of thrombosis in children.

The last article in topic, Wang et al. review the success of immediate surgical curettage and bone grafting for treatment of hemophilic pseudotumor of distal upper extremity in an adolescent with mild hemophilia A. As reviewed by the authors, hemophilic pseudotumor is a rather rare but serious complication in children with hemophilia, especially those with inhibitors. Hemophilic pseudotumor can be associated with significant morbidity. The report highlights the importance of this diagnosis (which can be unrecognized) and offers a surgical approach with close monitoring for their management. This case report serves as a valuable reminder of the challenges that may arise from limited resources, in contrast to previous articles reviewing issues that may be encountered with advancements in care. Unfortunately, unequal access to care remains a painful reality for most of the children with hemophilia across the world.
